# Simple thrombin-based method for eliminating fibrinogen interference in serum protein electrophoresis of haemodialysed patients

**DOI:** 10.11613/BM.2020.020705

**Published:** 2020-04-15

**Authors:** Anamarija Rade, Anamarija Đuras, Irena Kocijan, Patricija Banković Radovanović, Ana Turčić

**Affiliations:** 1 Medical biochemistry laboratory, General Hospital Varaždin, Varaždin, Croatia; 2 Department of transfusion medicine, General Hospital Pula, Pula, Croatia; 3 Department of Laboratory Diagnostics, University Hospital Centre Zagreb, Zagreb, Croatia

**Keywords:** fibrinogen, electrophoresis, central venous catheter, thrombin, interference

## Abstract

**Introduction:**

Serum samples of haemodialysed patients collected through vascular access devices, *e.g.* central venous catheter (CVC) can contain residual heparin, which can cause incomplete clotting and consequently fibrinogen interference in serum protein electrophoresis (SPE). We hypothesized that this problem may be overcome by addition of thrombin and aimed to find a simple thrombin-based method for fibrinogen interference removal.

**Materials and methods:**

Blood samples of 51 haemodialysed patients with CVC were drawn through catheter into Clot Activator Tube (CAT) and Rapid Serum Tube Thrombin (RST) vacutainers (Becton Dickinson, New Jersey, USA) following the routine hospital protocols and analysed with gel-electrophoresis (Sebia, Lisses, France). Samples were redrawn in the CAT tubes and re-analysed after being treated with thrombin using two methods: transferring CAT serum into RST vacutainer and treatment of CAT serum with fibrinogen reagent (Multifibren U, Siemens, Marburg, Germany).

**Results:**

Direct blood collection in RST proved to be slightly more efficient than CAT in removing the interfering band in beta fraction (CAT removed 6/51 and RST removed 12/51, P = 0.031). Transferring CAT serum into the RST vacutainer proved to be more efficient for subsequent removal of interfering band from CAT serum than the addition of fibrinogen reagent (39/45 *vs.* 0/45 samples with efficiently removed interfering band, P < 0.001).

**Conclusion:**

Fibrinogen interference caused by incomplete clotting because of residual heparin can be overcome by addition of thrombin. Transferring CAT serum into the RST vacutainer was the most efficient method.

## Introduction

Serum protein electrophoresis (SPE) is a simple, but important diagnostic tool for monitoring protein status in renal failure, hepatic disorders, chronic inflammation and pivotal for diagnosis and monitoring of patients with monoclonal gammopathies (*1*). Monitoring patients on haemodialysis is generally more problematic since preanalytically adequate serum samples are not easily obtained, and their samples often continue to coagulate for certain period of time beyond of what is expected. Heparin applied in therapeutic protocols of these patients hampers complete clotting and causes retention of residual fibrinogen in serum (*2*, *3*). Furthermore, haemodialysed patients, as well as those with chronic kidney disease, have significantly higher fibrinogen concentrations, carbon monoxide-mediated enhanced coagulation and diminished fibrinolytic vulnerability compared to healthy individuals (*4*, *5*).

Blood sampling in haemodialysed patients is often performed through vascular access devices (VAD) (*e.g.* the central venous catheter (CVC)) with the intention of sparing blood vessels from frequent punctures as venous access is required for medical treatment (*6*, *7*). Many researchers have investigated proper blood collection protocols for obtaining preanalytically acceptable samples through CVC (*8*-*10*). However, there is still a lack of consensus on the best method for removing heparin while avoiding major blood loss for the patient (*11*, *12*). Several protocols are suggested for reducing the problem of heparin contamination including the protocol in the relevant CLSI guidelines. Depending on the type of central line and requested analysis, VAD flushing and discarding of different volumes of blood is recommended prior to collecting blood sample for analysis (*13*-*15*). Although these guidelines are expected to be followed, their application in the routine setting is not always implemented. Discarding large amounts of blood every time the blood is drawn can cause iatrogenic anaemia, especially in haemodialysis patients that have particularly high risk of anaemia because of frequent blood draws and underlying renal disease. To prevent that, clinicians use different empirical techniques of blood collection from VAD, thereby enhancing the possibility that residual heparin from catheters causes the fibrinogen presence in serum due to incomplete clotting (*16*). Prevention of residual heparin in samples is at times unavoidable if the clinician’s efforts to spare patients are respected. In such cases the fibrinogen interference in SPE presents an analytical challenge for the laboratory.

Analytical interference in SPE is presented in the form of the monoclonal band located between beta and globulin fraction that is usually included in beta globulins causing a false positive result. This phenomenon of mimicking the monoclonal immunoglobulin is important to recognize in order to avoid unnecessary diagnostic procedures, clinical misinterpretation and inappropriate treatment (*17*, *18*). We hypothesized that thrombin-based method can overcome the inhibitory effect of heparin and remove fibrinogen by enforcing clot formation since thrombin is a principal enzyme in haemostasis. Thrombin, among other pro-coagulant actions, catalyses the clot formation by converting fibrinogen to fibrin. This activity is utilized in thrombin-based clot activator vacutainers intended for improving the test turnaround time and workflow efficiency (*21*). Our aim was to find a simple method for obtaining preanalytically correct samples for SPE in haemodialysed patients with CVC.

## Materials and methods

### Subjects

This observational cross-sectional study was conducted in the Medical biochemistry laboratory of General hospital Varaždin, Croatia, from September 2018 to February 2019. The study included 51 haemodialysed patients with CVC, 17 from Department of Nephrology and dialysis in General hospital Varaždin, 4 patients from Department of Nephrology in Special hospital for palliative care Novi Marof and 30 patients from Department of haemodialysis in General Hospital “Dr. Tomislav Bardek” Koprivnica. Blood samples were drawn in Clot Activator Tube (CAT) 6 mL and Rapid Serum Tube Thrombin (RST) 5 mL (Becton Dickinson, New Jersey, USA). Blood from the same patients was later redrawn in CAT tubes only in order to test further possible fibrinogen elimination protocols. Haemolytic, lipemic or icteric samples were not included in the study. The study was approved by the hospitals Ethics Committees and the written informed consent was obtained for all participating patients.

### Methods

Samples were collected right before haemodialysis the same way in all three hospitals following a routine hospital protocol. The blood was collected after high molecular heparin contained in the catheter was discarded by aspiration in a clean syringe followed by the subsequent aspiration of the additional 1 mL of blood that was also discarded. Blood was then collected through a catheter in a clean syringe and transferred in CAT and RST vacutainers. After blood collection, tubes were left for 30 minutes at room temperature and then centrifuged at 1780xg for 10 minutes. Serum was aliquoted in clean plastic tubes and stored for a maximum of 2 days at 2-8°C before analysis. Electrophoresis was performed on the Sebia Hydrasis analyser (Sebia, Lisses, France) using Hydragel 30 protein(e) agarose gel set. Aliquoted samples from both tubes were analysed side by side so distinct comparison could be made. Gels were inspected visually for the presence or absence of the interfering band in beta globulin fraction and results were presented as band absent - 0 and band present - 1.

The redrawn blood was collected through CVC following the same protocol as described above. Aliquoted CAT samples were then treated with thrombin in two separate ways. The first treatment method consisted of transferring 500 µL of CAT serum in an empty RST tube. The tube was inverted several times to ensure the complete dissolving of thrombin. The serum was then left at room temperature for 30 minutes and centrifuged at 1160xg for 10 minutes to settle fibrin clots in the sample. The second treatment method consisted of adding 10 µL of Multifibren U reagent (Siemens Healthcare, Marburg, Germany) in 100 µL of serum (total 0.5 IU of thrombin added, final concentration 4.55 IU/mL) and was left at room temperature for 30 minutes and centrifuged at 1160xg for 10 minutes. The volume of Multifibren U added to the sample was tested in a pilot study prior to this research (results not shown). Electrophoresis was performed for both treated aliquots along with the native serum, which served as a reference. The final sequence on the gel for each patient was as follows: 1. native serum (reference); 2. serum processed in RST vacutainer; 3. serum processed with Multifibren U. The intensity of electrophoretic patterns was measured by gel densitometry on Sebia Hydrasys 2 (Sebia, Lisses, France). Serum protein electrophoresis results for two tested methods were mutually compared to determine if sample manipulation influenced the protein fraction concentrations.

### Statistical analysis

Kolmogorov-Smirnov test was used to assess normal data distribution for results of electrophoretic fractions, since they did not follow normal distribution the data was expressed as median (interquartile range). Presence of interfering fibrinogen band in RST and CAT tubes were presented as number and ratio. Patients’ age was presented as median (minimum and maximum range). A dependent nominal variable, which described the difference between two sampling tubes regarding fibrinogen interference, was tested with a McNemar test. The difference between two methods for removal of the fibrinogen interference was tested with Cochran’s Q test followed by the Sheskin post hoc test. Differences in concentrations of protein fractions obtained by two methods for removing interference were tested with the Friedman test followed by the Conover *post hoc* test. P value < 0.05 was considered statistically significant. For statistical data processing MedCalc Statistical Software version 18.11.3 (MedCalc Software bvba, Ostend, Belgium) was used.

## Results

**Figure 1:**
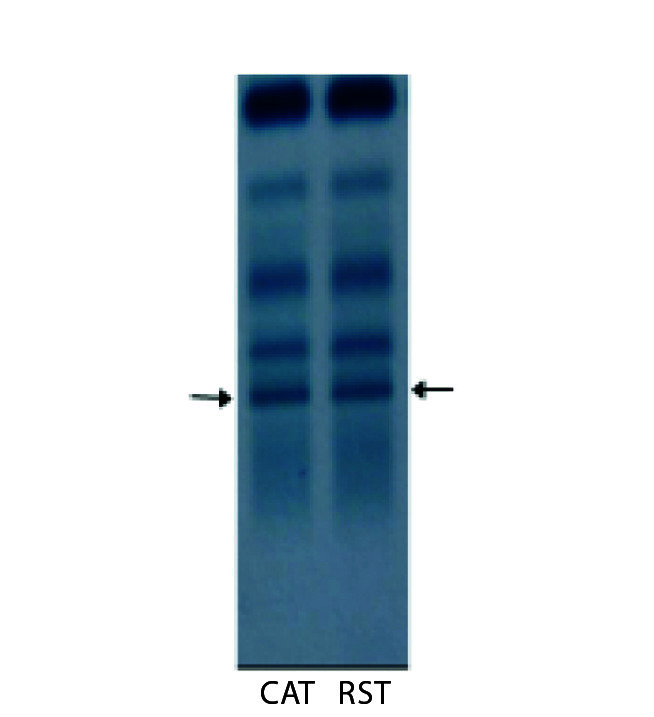
Comparison of serum protein electrophoresis of haemodialysed patient’s sample drawn in two different tubes regarding the monoclonal band in the beta fraction (marked with arrows) due to the fibrinogen interference. CAT - clot activator tube. RST - rapid serum tube.

The study included 51 patients on haemodialysis aged 67 (30 - 83). Fibrinogen interference was presented as a monoclonal band in the beta fraction of SPE (Figure 1). Interfering fibrinogen band was absent in 6/51 of CAT tubes *vs.* 12/51 of RST tubes (Table 1).

**Table 1:** Presence of monoclonal band in the beta fraction (fibrinogen interference) of SPE in samples of haemodialysed patients collected through CVC in CAT and RST tubes

**CAT serum** **(N = 51)**	**RST serum**		**P**
**Band absent**	**Band present**	
Band absent	6	0	0.031
Band present	6	39	
CAT - clot activator tube. CVC - central venous catheter. RST - rapid serum tube. SPE - serum protein electrophoresis. P < 0.05 was considered statistically significant.			

**Figure 2:**
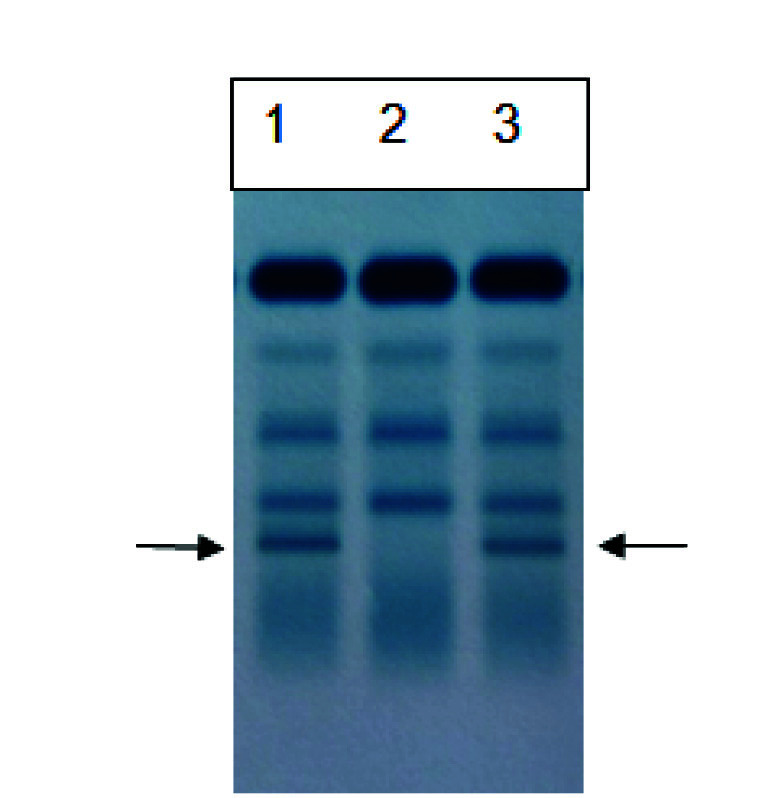
Comparison of serum protein electrophoresis of haemodialysed patient’s sample drawn through a central venous catheter into a CAT tube (reference) and treated with two thrombin-based methods regarding the efficiency in removing the fibrinogen interference (marked with arrows). 1 - Reference serum collected in clot activator tube (CAT). 2 - CAT serum transferred in rapid serum tube vacutainer. 3 - CAT serum treated with Multifibren U reagent.

When serum samples were redrawn in CAT tubes, 45/51 samples presented the interference. Efficiency in removing fibrinogen interference by treating the CAT sample with two thrombin-based methods was evaluated visually (Figure 2). Inspection of electrophoretic gel showed that transferring serum in a clean RST tube removed fibrinogen band in 39/45 samples, whereas adding Multifibren U didn’t remove interference in any of 45 samples (0/45, P < 0.001) compared to the corresponding reference sample (Table 2). Concentrations of protein fractions (CAT serum, CAT serum transferred to RST and CAT serum treated with Multifibren U) are presented in Table 3. Concentration of beta fractions in CAT serum transferred to RST tube was significantly lower compared to the reference sample (6.9 g/L *vs.* 11.2 g/L), but there was no quantitative difference in beta globulin fractions between CAT serum treated with Multifibren U and reference sample (11.3 g/L *vs*. 11.2 g/L).

**Table 2:** Efficiency of two thrombin-based methods for fibrinogen interference removal

**Method** **(N = 51)**	**Band absent**	**Band present**	**P**	**Sheskin** ***post hoc* test**
CAT tube (native serum) (1)	6	45	< 0.001	(2)/(1)(3)
500 µL CAT serum in RST tube (2)	45	6		
100 µL CAT Serum treated with 10 µL of Multifibren U (3)	6	45		
CAT - clot activator tube. RST - rapid serum tube. SPE - serum protein electrophoresis. P < 0.05 was considered statistically significant.				

**Table 3:** Comparison of protein concentration of all SPE fractions of reference sample (CAT serum) and two thrombin-based methods for removing fibrinogen interference

**Protein concentration** **(N = 51)**	**CAT serum** **(1)**	**500 µL of the CAT** **serum in RST (2)**	**CAT serum treated with Multifibren U (3)**	**P**	**Conover** ***post hoc* test**
Albumin, g/L	39.2 (36.5-42.4)	42.1 (38.4-44.7)	40.1 (37.3-42.7)	< 0.001	(2)/(1)(3)
alfa 1, g/L	1.8 (1.7-2.1)	1.9 (1.8-2.2)	1.9 (1.6-2.2)	< 0.001	(2)/(1)(3)
alfa 2, g/L	6.6 (6.2-7.6)	7.1 (6.3-8.0)	6.6 (6.1-7.6)	< 0.001	(2)/(1)(3)
beta, g/L	11,2 (9.4-12.8)	6.9 (6.1-8.3)	11.3 (9.4-12.6)	< 0.001	(2)/(1)(3)
gamma, g/L	7.5 (6.3-9.1)	9.4 (7.2-10.9)	7.2 (6.2-9.2)	< 0.001	(2)/(1)(3)
CAT - clot activator tube. RST - rapid serum tube. SPE- serum protein electrophoresis. SPE fractions were presented as median (interquartile range). P < 0.05 was considered statistically significant.					

## Discussion

The observation of the additional SPE band in serum of haemodialysis patients’ samples in our investigation is in concordance with the known possible heparin influence on sample quality (*22*). Strategies to avoid this interference described in the literature include fibrinogen removal from serum sample by using ethanol prior to electrophoresis as well as performing immunofixation with anti-fibrinogen antibodies to confirm that the band is indeed fibrinogen (*19*, *20*). However, this implies the additional expense due to the cost of antiserum and is time-consuming compared to enhancing thrombin concentration, which was investigated in our study. To the best of our abilities, we were unable to find other studies investigating the ways of dealing with this interference, especially in a sensitive population of haemodialysed patients.

Some authors have proposed to prepare the preanalytically acceptable sample of heparin-contained blood with powdered thrombin. In our investigation we tried to remove heparin’s interference by sampling in RST where we observed the statistically significant difference (P = 0.031), but considering that 6/51 samples in CAT tube were without interference in the first place, in only 6 of remaining 45 samples collected directly in RST tube the removing of monoclonal band from beta fraction was efficient comparing to corresponding sample in CAT tube.

The lack of clinically significant difference obtained in the results regarding the fibrinogen interference in electrophoresis between thrombin-based RST and non-thrombin-based CAT vacutainer indicates that thrombin contained in RST vacutainer is probably under-concentrated to overcome the inhibitory action of heparin and thus completely remove fibrinogen from the sample. As Dimeski *et al.* have showed, latent clotting in specimens collected in RST is continued in patients who have received a high concentration of anticoagulants (*23*). Therefore, we have further investigated the possibility of resolving this issue by enhancing thrombin concentration. It is difficult to estimate the adequate amount of thrombin considering that the actual concentration and form of thrombin in this vacutainer is unknown and represents the manufacturer’s proprietary data.

The addition of thrombin in form of Multifibren U solution was operatively more demanding. Complete clotting should be achieved with as little reagent volume as possible to avoid consequent dilution of the sample. In order to achieve the best results, the amount of Multifibren U added was tested in a pilot study prior to this research and it was decided to be the amount that was the most successful in diminishing the interference without distorting other SPE fractions (results not shown). Despite all efforts, the method was not proven efficient since there was no effect on removal of fibrinogen interference and other protein fractions. This might be explained by the insufficient specific activity of thrombin in Multifibren U, like in other commercial reagents that ineffectively clot fibrinogen (*24*). The addition of more thrombin would possibly give better results, but it could cause differences in electrophoretic fractions due to the sample dilution.

Therefore, we have opted to transfer CAT serum in a clean RST tube. This approach was far more efficient in removing interference than serum treated with Multifibren U.

The manipulation with serum could cause problem due to the specific relations between protein fractions in SPE which proportions are interdependent, thus, removing fibrinogen from the beta fraction could cause change on protein concentrations of other fractions. After serum was transferred in RST all fractions showed to be statistically different, but only difference in beta fraction was clinically significant and resulted in visual removal of dense band in electrophoretic pattern. These findings confirmed that the method of transferring a serum aliquot to the thrombin-containing RST vacutainer efficiently removed fibrinogen interference in beta fraction without clinically affecting other protein fractions. A practical implication of our findings is that serum samples with residual heparin can be used for the SPE after proper heparin removal avoiding the data misinterpretation. We are fully aware that sample manipulation does not replace properly collected sample and obtained results should not be reported, but it could be helpful especially in samples of haemodialysed patients to clarify if the presence of monoclonal band in beta globulin fraction is a result of a preanalytically poor quality sample and further clinical or laboratory investigation is required. In cases where interference is eliminated partially or fully, the laboratory can require a new, properly collected sample. This approach is a promising practical alternative to other methods of selective fibrinogen interference elimination known from the literature and can potentially be used to avoid reporting erroneous results which can lead to further unnecessary testing (*18*, *19*, *25*-*27*).

Clear limitation of this study is the non-compliance with the respective CLSI guidelines on sampling from VAD in the institutions included in our study (*13*).

The results of our investigation confirm that sampling of haemodialysed patient’s blood trough CVC in thrombin-based RST vacutainer and addition of thrombin in the form of fibrinogen reagent cannot remove fibrinogen interfering in serum protein electrophoresis. Instead, a much higher ratio of thrombin to sample is necessary for overcoming the inhibitory effect of heparin. Transferring a serum aliquot in a clean RST tube is a simple method for efficiently eliminating or at least reducing fibrinogen in a routine setting.
